# RhoB blockade selectively inhibits autoantibody production in autoimmune models of rheumatoid arthritis and lupus

**DOI:** 10.1242/dmm.029835

**Published:** 2017-11-01

**Authors:** Laura Mandik-Nayak, James B. DuHadaway, Jennifer Mulgrew, Elizabeth Pigott, Kaylend Manley, Summer Sedano, George C. Prendergast, Lisa D. Laury-Kleintop

**Affiliations:** 1 Lankenau Institute for Medical Research, Wynnewood, PA 19096, USA; 2 Department of Pathology, Anatomy and Cell Biology, Sidney Kimmel Medical College and Kimmel Cancer Center, Thomas Jefferson University, Philadelphia, PA 19107, USA

**Keywords:** Rho GTPases, Autoantibodies, Rheumatoid arthritis, K/BxN, MRL/*lpr*, Systemic lupus erythematosus

## Abstract

During the development of autoimmune disease, a switch occurs in the antibody repertoire of B cells so that the production of pathogenic rather than non-pathogenic autoantibodies is enabled. However, there is limited knowledge concerning how this pivotal step occurs. Here, we present genetic and pharmacological evidence of a positive modifier function for the vesicular small GTPase RhoB in specifically mediating the generation of pathogenic autoantibodies and disease progression in the K/BxN preclinical mouse model of inflammatory arthritis. Genetic deletion of RhoB abolished the production of pathogenic autoantibodies and ablated joint inflammation in the model. Similarly, administration of a novel RhoB-targeted monoclonal antibody was sufficient to ablate autoantibody production and joint inflammation. In the MRL/*lpr* mouse model of systemic lupus erythematosus (SLE), another established preclinical model of autoimmune disease associated with autoantibody production, administration of the anti-RhoB antibody also reduced serum levels of anti-dsDNA antibodies. Notably, the therapeutic effects of RhoB blockade reflected a selective deficiency in response to self-antigens, insofar as RhoB-deficient mice and mice treated with anti-RhoB immunoglobulin (Ig) both mounted comparable productive antibody responses after immunization with a model foreign antigen. Overall, our results highlight a newly identified function for RhoB in supporting the specific production of pathogenic autoantibodies, and offer a preclinical proof of concept for use of anti-RhoB Ig as a disease-selective therapy to treat autoimmune disorders driven by pathogenic autoantibodies.

## Abstract (precis)

**Editors' choice:** The small GTPase RhoB participates in the development of an autoimmune response, specifically by influencing the production of pathogenic autoantibodies.

## INTRODUCTION

Evidence shows that nonpathogenic autoantibodies accumulate in individuals years before the emergence of symptomatic autoimmune disease. In considering this phenomenon, a fundamental issue is identifying the factors dictating a switch in production from nonpathogenic to pathogenic autoantibodies, which is a critical step in subsequent presentation of a frank clinical disorder. Rheumatoid arthritis (RA) is an example of a debilitating autoimmune disease characterized by chronic inflammation of the synovial joints (Feldmann et al., 1996). Erosion and remodeling of the joint cartilage and bone results from infiltration of inflammatory cells, increased levels of synovial fluid and proinflammatory cytokines, as well as complement deposition (Feldmann et al., 1996). The importance of B cells in driving the initiation of this autoimmune response is suggested by the correlation of autoantibody titers with disease progression and clinically by the improvement of disease upon B-cell depletion (De Vita et al., 2002). Despite the great understanding of the later inflammatory stages of autoimmune diseases such as RA, little is known about the factors involved in controlling the production of disease-causing autoantibodies. Although great progress has been made in therapeutic management, current therapies for autoimmune disease do not selectively target autoreactive cells but are directed against general inflammatory processes. Even with the most successful therapies, there remains heightened treatment risks and side effects that include the development of cancer and serious infections in patients (reviews include Lateef and Petri, 2010; Singh, 2016; Tanaka, 2016; Woodworth and den Broeder, 2015).

RhoB is a stress-inducible member of the Rho family of small GTPases that influence membrane dynamics and the actin cytoskeleton (Biro et al., 2014; Hall, 2012; Murali and Rajalingam, 2014; Thumkeo et al., 2013; Wheeler and Ridley, 2007). Unlike many other members of this family, RhoB is dispensable for development and normal physiology but critical for stress responses that modify disease processes (Chen et al., 2006; Fritz et al., 1995; Huang et al., 2007; Liu et al., 2001; Prendergast, 2001; Adini et al., 2003; Bravo-Nuevo et al., 2011b). Although RhoB has been studied little in adaptive immunity, the canonical Rho proteins Rac1, Cdc42 and RhoA, as well as their regulators and effectors, are recognized widely to participate in B- and T-cell development, activation, proliferation, differentiation and migration (Biro et al., 2014; Reedquist and Tak, 2012; Saoudi et al., 2014; Tybulewicz and Henderson, 2009). Similarly, a variety of studies have implicated Rho GTPases in autoimmunity. Statins have been suggested to exert their anti-inflammatory effects in arthritic joints by modulating the post-translational prenylation and membrane binding of Rho small GTPases (reviewed in Reedquist and Tak, 2012). In the model of collagen-induced arthritis (CIA), Rac1 blockade with an inhibitory peptide reduced autoantibody levels and disease severity (Abreu et al., 2010). In human fibroblast-like synoviocytes isolated from RA patients, Rac1 inhibition was sufficient to reduce their proliferation and invasiveness (Chan et al., 2007). Similarly, a RhoA-targeted approach inhibited the migration, adhesion and invasion of these fibroblast-like synoviocytes (Xiao et al., 2013). Collectively, such studies suggest roles for Rho GTPases in normal immunity and autoimmunity.

In this study, we show how RhoB acts as a positive modifier of disease in the development of pathogenic autoantibodies required to drive arthritis in K/BxN mice, an established preclinical model of autoimmune disease. K/BxN mice spontaneously develop a joint-specific autoimmune disease that shares many similarities with human RA, including autoantibody production, inflammatory cytokine expression and joint pathology (Korganow et al., 1999; Kouskoff et al., 1996). Disease in this model is mediated by pathogenic autoantibodies directed against the glycolytic enzyme glucose-6-phosphate isomerase (GPI) and is dependent on both T and B cells (Korganow et al., 1999; Kouskoff et al., 1996; Maccioni et al., 2002; Matsumoto et al., 1999). Using this model, we explored the effects of genetic RhoB deletion along with a unique approach to target this protein's function through the development of an anti-RhoB immunoglobulin (Ig). Through this dual approach based on genetic or pharmacological targeting, we obtained evidence that RhoB is dispensable for normal immune function to a model antigen but important for pathogenic autoantibody production and joint inflammation. In a second preclinical model of autoimmunity, the MRL/*lpr* model of systemic lupus erythematosus (SLE), administration of anti-RhoB Ig also selectively diminished production of anti-double-stranded-DNA (anti-dsDNA) autoantibodies. Together, our findings suggest the utility of RhoB as a molecular probe to explore fundamental questions about the development of self versus non-self and the mechanisms controlling the emergence of autoimmune disorders.

## RESULTS

### Anti-RhoB Ig inhibits autoantibody production and attenuates disease in the K/BxN model of inflammatory arthritis

During a long-standing project to produce monoclonal antibodies (mAbs) that could specifically recognize RhoB versus other family members, we made the serendipitous observation that hybridomas would initially secrete anti-RhoB Ig and then consistently arrest production. Only by generating a fusion partner derived from RhoB-deficient splenocytes and fusing to splenocytes from immunized RhoB-deficient mice were we able to generate stable anti-RhoB-Ig-secreting hybridoma cell lines that specifically recognized RhoB and not RhoA, Cdc42 or Rac1 (Fig. S1). This, together with studies suggesting the importance of the GTPases RhoA, Cdc42 and Rac1 in normal B- and T-cell development and activation (Biro et al., 2014; Reedquist and Tak, 2012; Saoudi et al., 2014; Tybulewicz and Henderson, 2009), led us to hypothesize that RhoB may be necessary for antibody production. Based upon this line of reasoning, we asked whether administering the anti-RhoB Ig might shut down antibody secretion in a preclinical mouse model of autoimmune disease, where autoantibody production serves as a pathogenic driver, as well as in the context of an immune response to a model antigen.

**Fig. 1.**
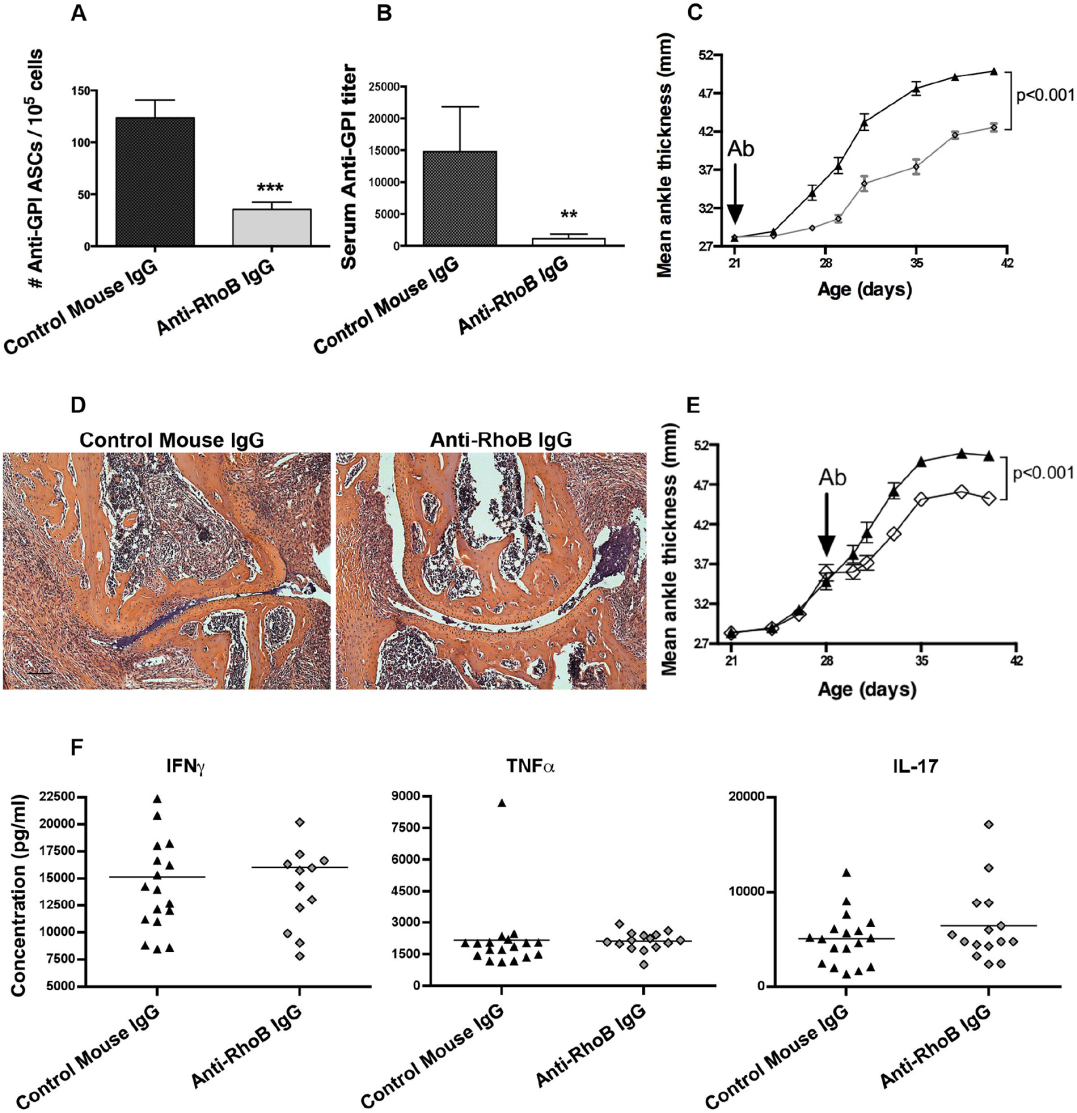
**Anti-RhoB Ig inhibits autoantibody production and attenuates arthritis.** K/BxN mice were treated with 500 µg anti-RhoB Ig at (A-D,F) 21 days or (E) 28 days of age. (A) The number of anti-GPI ASCs was measured by ELISpot at the termination of the experiment (6 weeks of age). Data show means±s.e.m. for *n*=19 control-Ig- and *n*=16 anti-RhoB-Ig-treated mice. (B) Titers of anti-GPI Ig in the serum were also measured at 6 weeks of age by ELISA. Data show means±s.e.m. for *n*=8 mice of each treatment group. (C,E) Joint inflammation was measured by the change in ankle thickness. Data show means±s.e.m. for (C) *n*=12 control Ig (▲) and *n*=14 anti-RhoB Ig (◊), and (E) *n*=9 control Ig (▲) and *n*=10 anti-RhoB Ig (◊)-treated mice. (D) Metatarsal joint harvested at 6 weeks of age stained with H&E. Representative sections from a total of *n*=5 mice for each treatment group are shown. Scale bar: 100 µm. (F) K/BxN mice were treated with 500 µg anti-RhoB Ig at 21 days of age. Three weeks later, the LNs draining the arthritic joints were harvested, and the isolated cells were stimulated with PMA+ionomycin overnight. Inflammatory cytokines were measured in culture supernatants using the cytometric bead array and flow cytometry. Each symbol depicts an individual mouse (control Ig, *n*=18; anti-RhoB Ig, *n*=15) with the mean indicated by a solid bar. ***P<0.001. **P<0.01.

To determine whether anti-RhoB Ig could inhibit autoantibody production, we employed the established K/BxN murine model of arthritis that is mediated by high titers of anti-GPI autoantibodies. After administering a single intraperitoneal (i.p.) injection of anti-RhoB Ig or control murine Ig before disease onset, K/BxN mice were analyzed for the number of anti-GPI-autoantibody-secreting cells (ASCs) in the joint-draining lymph nodes (LNs) and titers of anti-GPI Ig in the serum. Compared to treatment with control Ig, administration of the anti-RhoB Ig significantly lowered the number of anti-GPI ASCs in the draining LNs (Fig. 1A). Additionally, dosing with anti-RhoB reduced the titer of anti-GPI autoantibody in the serum (Fig. 1B), but did not alter the dominance of the IgG_1_ isotype (data not shown). In the K/BxN model, the level of anti-GPI Ig is correlated tightly with disease progression (Korganow et al., 1999; Mandik-Nayak et al., 2002). Therefore, the anti-RhoB-Ig-treated mice were also analyzed to determine whether a reduction in joint inflammation would be observed. Similarly to untreated mice, control-Ig-treated mice developed arthritis starting at 4 weeks of age that progressed rapidly over the next 2 weeks (Fig. 1C). In contrast, anti-RhoB-Ig-treated mice exhibited a significant attenuation in arthritis development, both at the time of onset and in terms of overall severity (Fig. 1C). Consistent with the decrease in joint inflammation, histological examination of ankles from anti-RhoB-Ig-treated mice confirmed a reduction in the severity of arthritis, with fewer infiltrating inflammatory cells and minimal synovial thickening and erosion of the cartilage and bone (Fig. 1D), suggesting an attenuation of the acute inflammatory phase characteristic of this model. Importantly, anti-RhoB Ig remained effective at attenuating arthritis even when it was administered after the onset of arthritis, suggesting its use both prophylactically and therapeutically (Fig. 1E).

### No apparent effect of anti-RhoB Ig on inflammatory cytokines or lymphoid cell repertoire

The inflammatory cytokines TNFα, IFNγ, IL-6 and IL-17 have been shown to play crucial roles in the development of arthritis in the K/BxN model (Ji et al., 2002; Koenders et al., 2006). Other cytokines, including IL-4, IL-10 and MCP-1, have also been implicated in disease development or progression (Ohmura et al., 2005; Scott et al., 2009). To determine whether anti-RhoB Ig affected cytokine production, we measured levels of these and other cytokines in the joint-draining LNs from treated mice. As expected, we observed high levels of TNFα, IFNγ and IL-17 in control-Ig-treated mice; however, treatment with anti-RhoB Ig did not affect these levels (Fig. 1F), suggesting that the LN cells from anti-RhoB-Ig-treated mice retained the ability to make equivalent amounts of cytokines in response to phorbol myristate acetate (PMA)/ionomycin. Levels of IL-4, IL-5, IL-6, IL-9, IL-10, IL-13, MCP-1, MIP-1α and MIP-1β were also similar in animals treated with anti-RhoB Ig or control Ig (Fig. S2). Additionally, an analysis of the lymphoid cell repertoires in the spleen, LNs, thymus, bone marrow and peritoneal cavity did not reveal significant differences in the frequency of the cell types when K/BxN mice were dosed with anti-RhoB Ig (Fig. S3). Collectively, these data are suggestive that anti-RhoB Ig is not generally immunosuppressive.

### RhoB-deficient KRN.g7 mice exhibit decreased arthritis

**Fig. 2.**
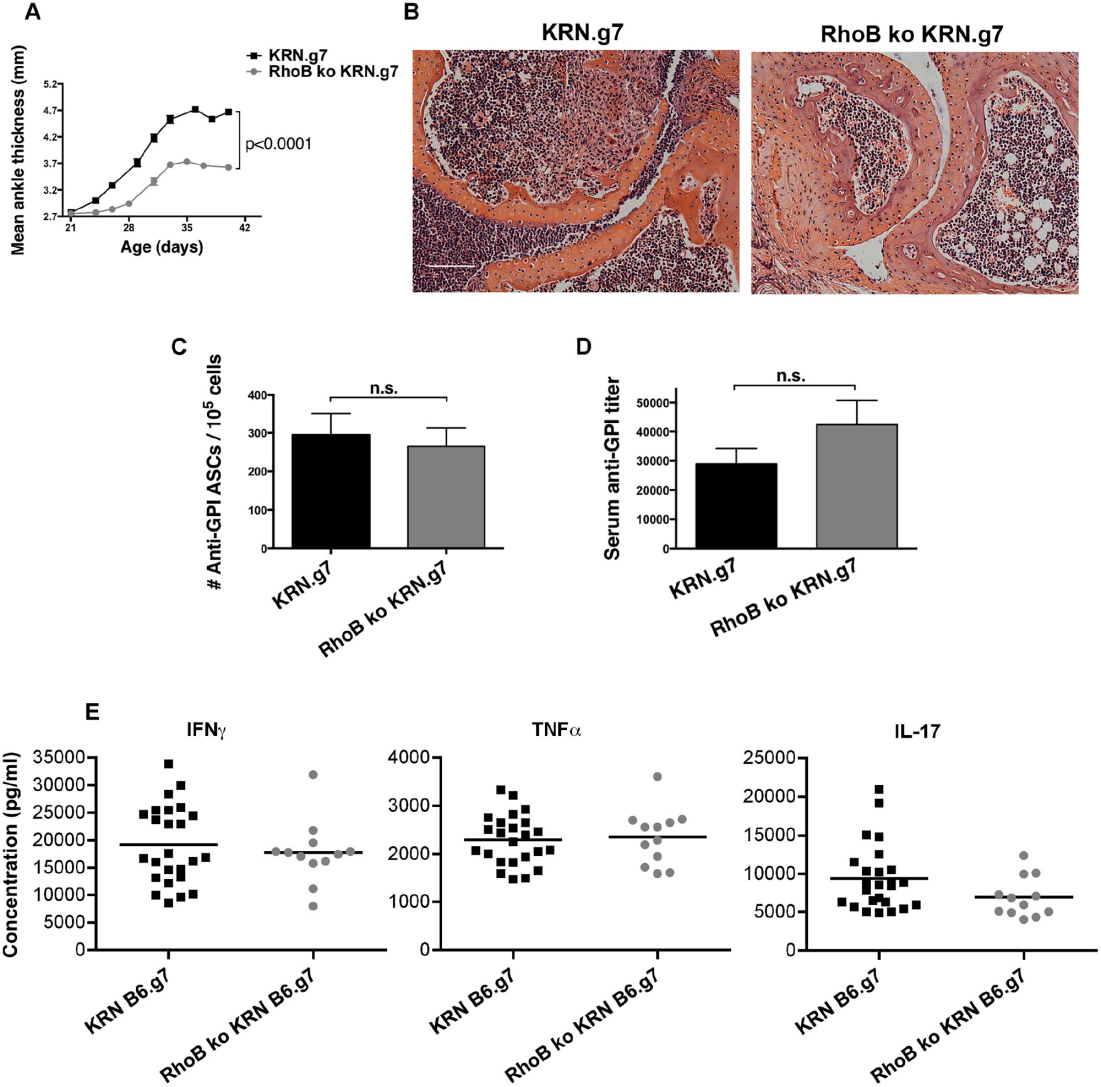
**Arthritis, but not autoantibody level, is attenuated in RhoB ko mice.** The *RhoB* ko allele was crossed into the KRN model on the C57BL/6 background. (A) Joint inflammation was determined by measuring ankle thickness. Data show means±s.e.m. for *n*=25 KRN.g7 and *n*=14 RhoB ko KRN.g7 mice. (B) Metatarsal joint harvested at 6 weeks of age stained with H&E. Representative sections from a total of *n*=5 mice for each treatment group are shown. Scale bar: 100 µm. At 6 weeks of age, (C) the number of anti-GPI ASCs was measured by ELISpot; data show means±s.e.m. for *n*=15 KRN.g7 and *n*=17 RhoB ko KRN.g7 mice; n.s., not significant; and (D) titers of anti-GPI Ig in the serum were measured by ELISA. Data show means±s.e.m. for *n*=23 KRN.g7 and *n*=11 RhoB ko KRN.g7 mice. (E) At 6 weeks of age, LNs draining the arthritic joints were harvested and the isolated cells were stimulated with PMA+ionomycin overnight. Inflammatory cytokines were measured in culture supernatants using the cytometric bead array and flow cytometry. Each symbol depicts an individual mouse with the mean indicated by a solid bar (KRN B6.g7, *n*=25; RhoB ko KRN B6.g7, *n*=12).

To genetically evaluate the relevance of RhoB in the autoimmune response, we crossed the *RhoB* null allele into the KRN model on a pure C57BL/6 background (identified as KRN.g7) to generate mice with wild-type (wt) or homozygous-null [knockout (ko)] genotypes. As demonstrated previously, wt KRN.g7 mice develop arthritis and produce autoantibodies with similar kinetics to those in K/BxN mice (Kouskoff et al., 1996). Consistent with results from anti-RhoB-antibody-treated mice, development of arthritis in RhoB ko KRN.g7 mice was delayed and attenuated, but not fully ablated, relative to wt KRN.g7 mice (Fig. 2A). Histological examination of ankles from RhoB ko KRN.g7 mice confirmed a reduction in arthritis as measured by reduced synovial expansion, inflammatory cell infiltrates, and cartilage and bone erosion (Fig. 2B). However, unlike mice treated with anti-RhoB Ig, neither the number of ASCs nor serum autoantibody titers were reduced in RhoB ko KRN.g7 animals (Fig. 2C,D). Also consistent with the results of anti-RhoB-Ig administration, levels of the inflammatory cytokines TNFα, IFNγ and IL-17 were similar between RhoB-deficient and wt KRN.g7 mice (Fig. 2E), as were the levels of IL-4, IL-5, IL-6, IL-9, IL-10, IL-13, MCP-1, MIP-1α and MIP-1β (Fig. S4).

### Anti-RhoB Ig does not alter the arthritis of RhoB ko KRN.g7 mice

**Fig. 3.**
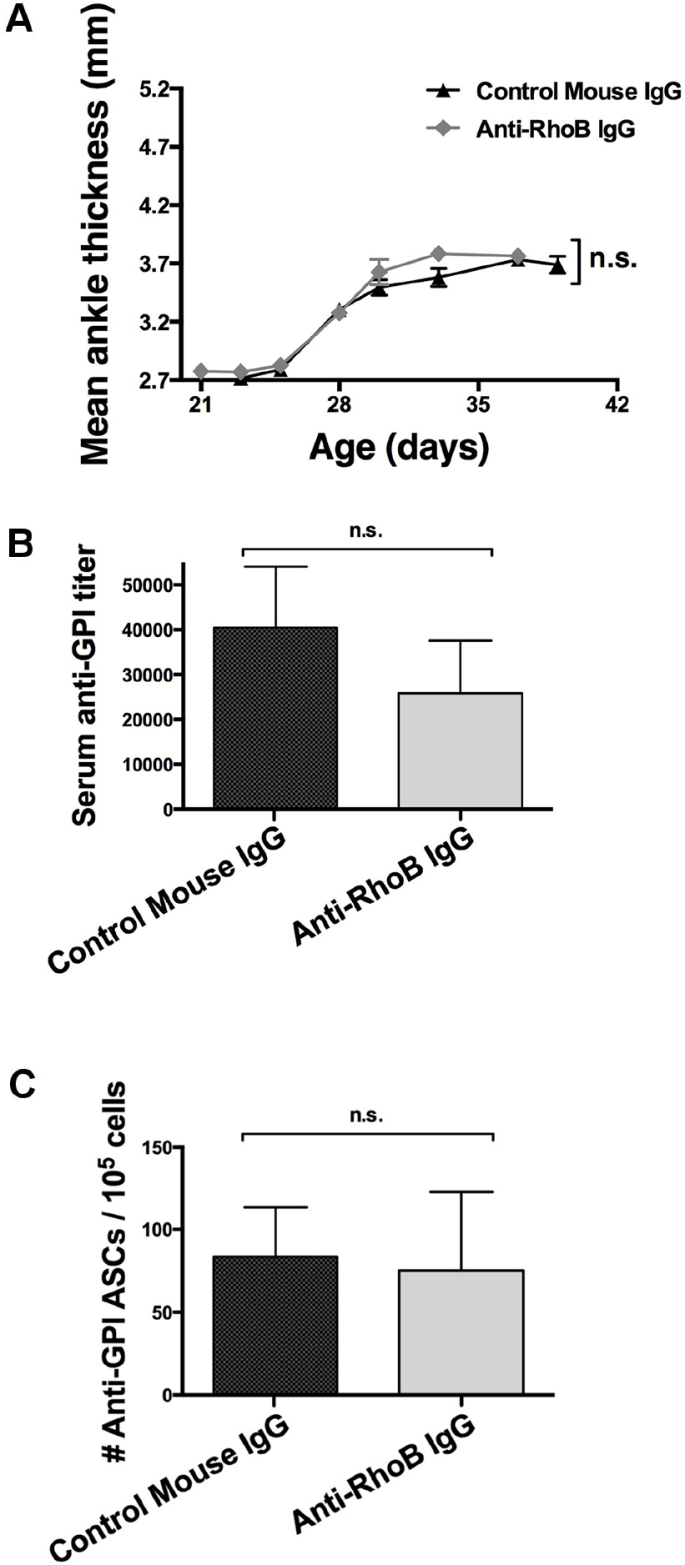
**Anti-RhoB Ig has no effect in RhoB ko arthritic mice.** RhoB ko KRN.g7 mice were treated with 500 µg anti-RhoB Ig at 21 days of age. Data show means±s.e.m. for *n*=5 control-Ig- and *n*=5 anti-RhoB-Ig-treated mice. n.s., not significant. (A) Joint inflammation was measured by the change in ankle thickness. At 6 weeks of age, (B) titers of anti-GPI Ig in the serum were measured by ELISA and (C) the number of anti-GPI ASCs was measured by ELISpot.

Overall, the pattern in the disease phenotype of ko and wt KRN.g7 was similar, if not identical, to that seen in mice treated with anti-RhoB Ig versus control Ig. To validate RhoB as the target and establish whether RhoB was required for the effect of anti-RhoB Ig, we evaluated arthritis and autoantibody production in RhoB ko KRN.g7 mice, in which arthritis is diminished compared with KRN.g7 mice but not fully absent (Fig. 2A,B). Administration of the anti-RhoB Ig did not further reduce the level of joint inflammation produced by genetic ablation (Fig. 3A). Likewise, the number of anti-GPI ASCs (Fig. 3B) and titers of anti-GPI Ig in the serum (Fig. 3C) were similar in ko KRN.g7 mice treated with either anti-RhoB Ig or control Ig. As an important control we confirmed that anti-RhoB Ig attenuated arthritis severity in wt KRN.g7 (Fig. S5). These data provide *in vivo* evidence that RhoB is crucial for the anti-arthritic effects of anti-RhoB Ig.

### RhoB-deficient mice do not produce pathogenic autoantibodies in serum

**Fig. 4.**
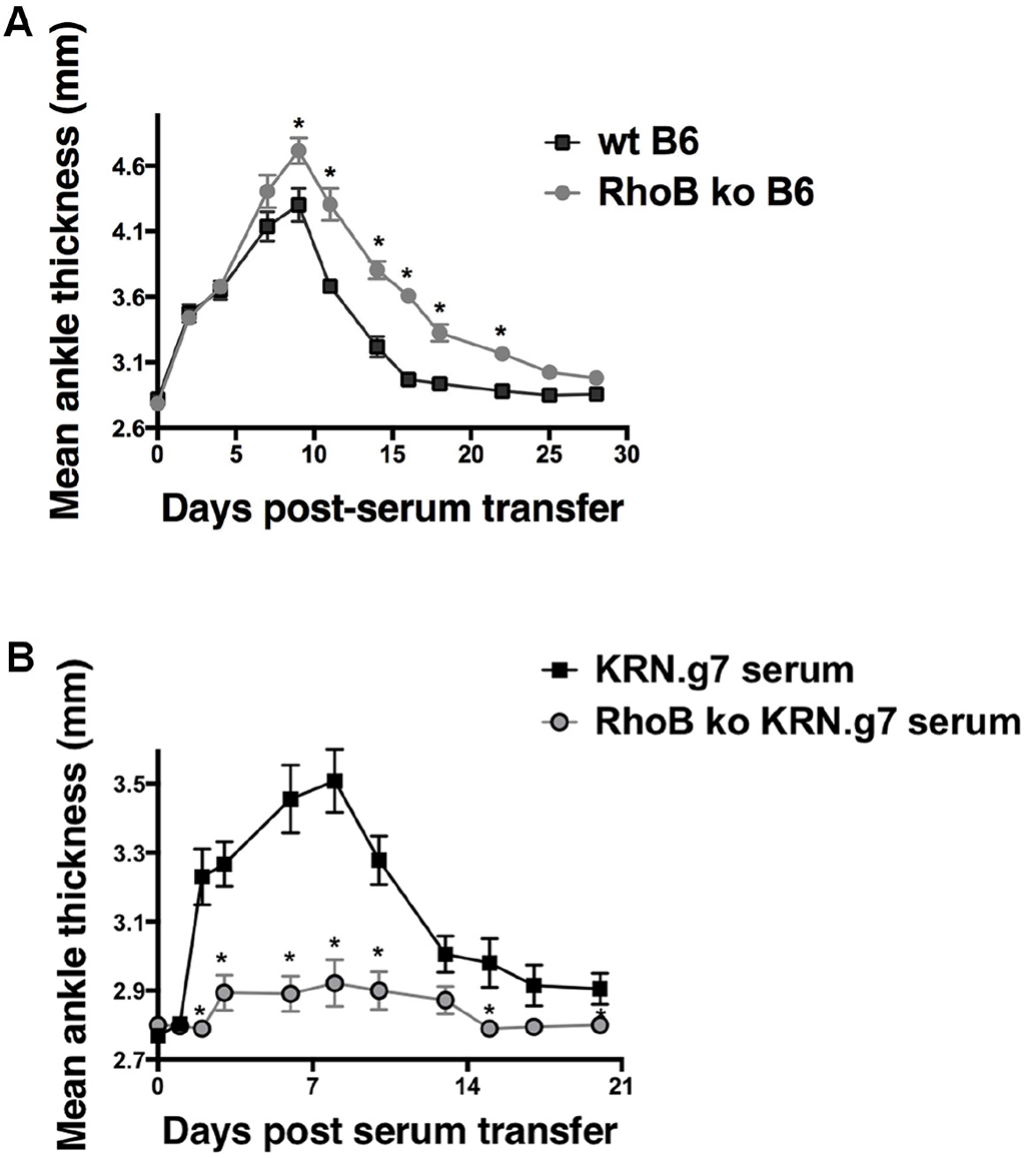
**Serum from RhoB ko KRN.g7 mice does not induce arthritis in a serum-transfer model.** (A) Pooled serum from K/BxN mice was injected into wt or RhoB ko C57BL/6 mice. Joint inflammation was measured by the change in ankle thickness. Data show means±s.e.m. from a representative experiment of two, with *n*=5 mice for each group. (B) Pooled serum from 6-week-old KRN.g7 or RhoB ko KRN.g7 mice was equalized for anti-GPI titer and injected into C57BL/6 recipient mice. Data show mean ankle thickness±s.e.m. from *n*=8 mice for each group. *P<0.05.

As mentioned above, in this arthritis model the level of anti-GPI Ig is correlated tightly with disease progression (Korganow et al., 1999; Mandik-Nayak et al., 2002). Therefore, our results showing that autoantibody levels were not reduced in RhoB ko KRN.g7 mice, but arthritis was attenuated, seemed paradoxical. Arthritis development in K/BxN mice can be divided into two stages: an initiation stage, dependent on B-cell activation and production of autoantibodies, followed by an effector stage involving downstream inflammatory mediators, including macrophages, mast cells and neutrophils (Korganow et al., 1999; Lee et al., 2002; Solomon et al., 2005; Wipke and Allen, 2001). Thus, one possible explanation for the reduced arthritis observed in the RhoB ko KRN.g7 mice, despite their high autoantibody levels, was that RhoB was critical at the effector stage of arthritis occurring after autoantibody production. To determine whether RhoB ko mice were able to develop arthritis, we made use of the serum-transfer model of arthritis (Korganow et al., 1999). In this model, arthritis is passively transferred to naive recipient mice by injecting serum from arthritic mice, thereby experimentally separating autoantibody production during the initiation stage from the downstream effector response. Serum from arthritic K/BxN mice induced robust arthritis in wt C57BL/6 recipients within 2 days, and disease severity increased until day 10 and resolved after 2 weeks. RhoB ko C57BL/6 recipient mice also developed arthritis within the same timeframe, although their arthritis was slower to resolve (Fig. 4A). These results suggest that the reduced arthritis in the RhoB ko KRN.g7 mice was not due to a defect in the downstream effectors, but point to a role for RhoB in the initiation phase.

Another possibility is that the anti-GPI Ig produced in the RhoB ko KRN.g7 animals, although similar in titer to wt, was not able to induce arthritis. Indeed, studies using monoclonal anti-GPI Igs cloned from arthritic K/BxN mice have shown that only a subset of anti-GPI Igs is able to induce disease (Maccioni et al., 2002). Therefore, RhoB could play a role in programming pathogenic autoantibody production elicited in the initiation phase. To determine whether the anti-GPI Ig detected in the serum of RhoB ko animals was pathogenic, we again made use of the serum-transfer model with serum from wt or RhoB ko KRN.g7 mice and recipient wt C57BL/6 naïve mice. If RhoB was critical for pathogenic autoantibody production, then serum transferred from the ko KRN.g7 mice should not induce arthritis, even though it contained anti-GPI autoantibodies. Strikingly, we found that serum transfer from RhoB ko KRN.g7 mice failed to induce arthritis in wt recipient mice, compared to serum transferred from wt KRN.g7 mice, which caused disease (Fig. 4B). Together, these data suggest that RhoB is essential for programming the pathogenic character of the autoantibody repertoire mediating arthritis in this model system, potentially acting as a unique molecular determinant in autoimmune disease. An interesting path to explore in the future will be to characterize the repertoire of arthritogenic Igs after anti-RhoB dosing and in RhoB ko KRN.g7 mice, similar in design to that reported by Maccioni and coworkers (Maccioni et al., 2002) in illuminating differences in epitopes targeted, affinity maturation and Ig gene usage.

### RhoB targeting does not disrupt normal immune-cell repertoire or function

**Fig. 5.**
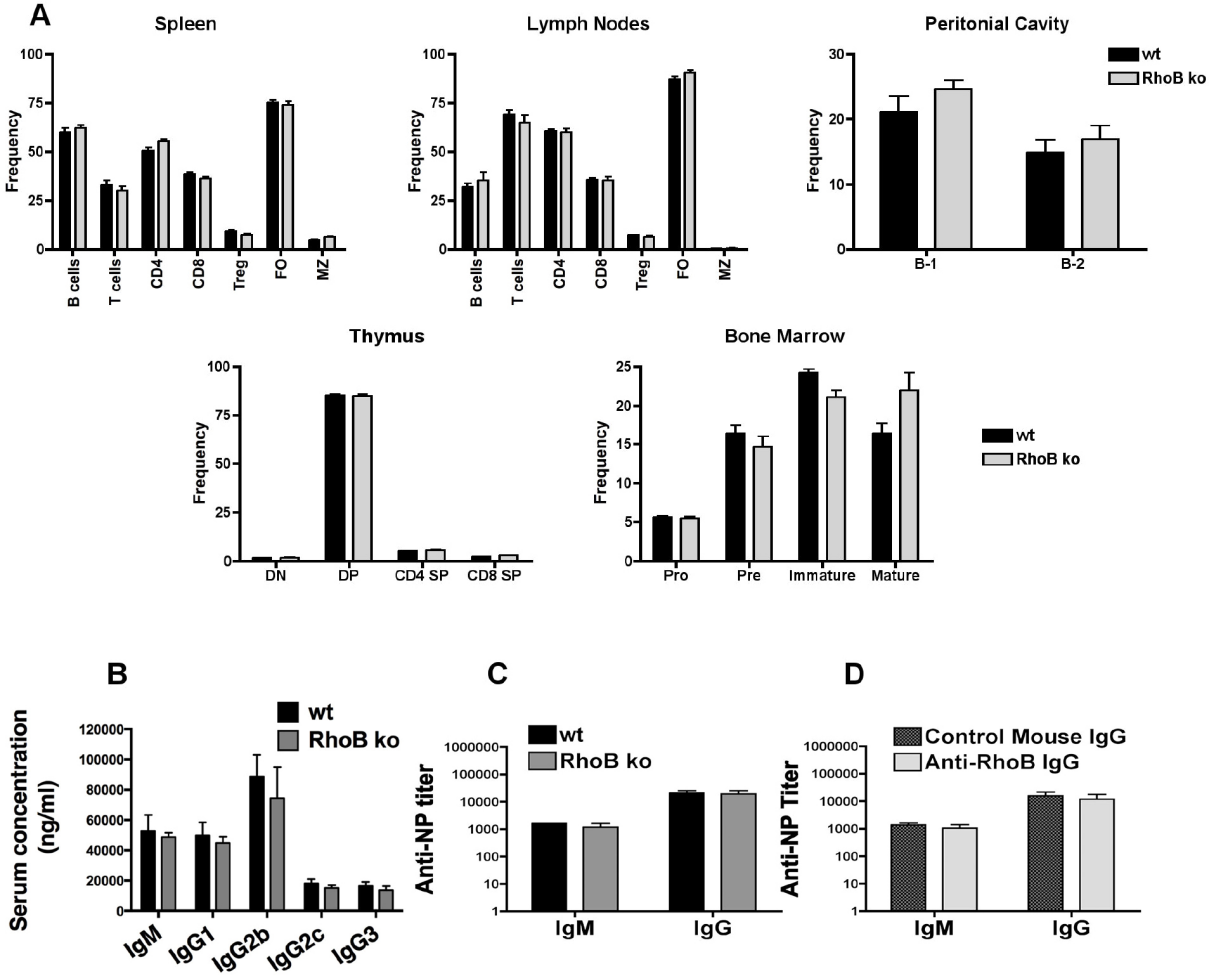
**Targeting RhoB does not affect immune-cell repertoire or function in either RhoB ko or anti-RhoB-Ig-treated C57BL/6** **mice.** (A) The frequency of individual lymphoid populations in wt or RhoB ko C57BL/6 mice was measured by flow cytometry. Data show means±s.e.m., *n*=6 mice of each genotype. Gating strategies are shown in Fig. S6B. (B) The amount of serum Ig in wt or RhoB ko C57BL/6 mice was measured by ELISA. *n*=6 mice of each genotype. (C) wt and RhoB ko C57BL/6 mice or (D) control-Ig- and anti-RhoB-Ig-treated C57BL/6 mice were immunized with 100 µg NP-KLH. Serum was harvested 10 days later and serum anti-NP titers were measured by ELISA. Data show means±s.e.m. for *n*=5 mice of each group. All analyses were performed three times.

The data above suggested that RhoB affected a specific feature of antibody production related to autoreactive pathogenicity and as such may not affect other non-autoreactive immune functions. To date, little to no data have been reported describing deficiencies in the immune system or immune responses for mice that are homozygotic for the *RhoB* null allele (Bravo-Nuevo et al., 2011a). Therefore, we evaluated the requirement of RhoB in general immune function by comparing immune-cell repertoires, basal serum Ig levels and immunization responses of RhoB ko and wt C57BL/6 mice (Fig. 5). No significant differences were observed in the frequency or absolute numbers of: pro-B, pre-B, immature B and mature B cells in the bone marrow; double-negative, double-positive, and CD4 and CD8 single-positive T cells in the thymus; B-1 or B-2 B cells in the peritoneal cavity; or CD4^+^ T cells, CD8^+^ T cells, regulatory T cells, and follicular and marginal zone B cells in the spleen and LNs (Fig. 5A; Fig. S6A). Similarly, we observed no differences in levels of serum IgM, IgG_1_, IgG_2b_, IgG_2c_ and IgG_3_ between ko and wt C57BL/6 mice (Fig. 5B). To determine whether RhoB was critical for an induced antibody response, we compared the phenotype of RhoB ko and wt C57BL/6 mice challenged by injection with the model antigen NP-KLH (4-hydroxy-3-nitrophenyl acetyl-keyhole lympet hemocyanin), and analyzed mice for serum titers of anti-NP IgM and IgG (Fig. 5C). A similar experiment was conducted in which wt C57BL/6 mice were administered anti-RhoB Ig or control Ig using the same conditions as above (Fig. 5D). In both settings, we observed no significant difference in the generation of low- or high-affinity IgM and IgG responses to NP-KLH immunization (data included for anti-NP3 titers). Taken together, these data led us to conclude that normal immune-cell development and response to immunization is comparable between RhoB-targeted and wt mice.

### Anti-RhoB Ig inhibits autoantibody production in the MRL/*lpr* model of SLE

**Fig. 6.**
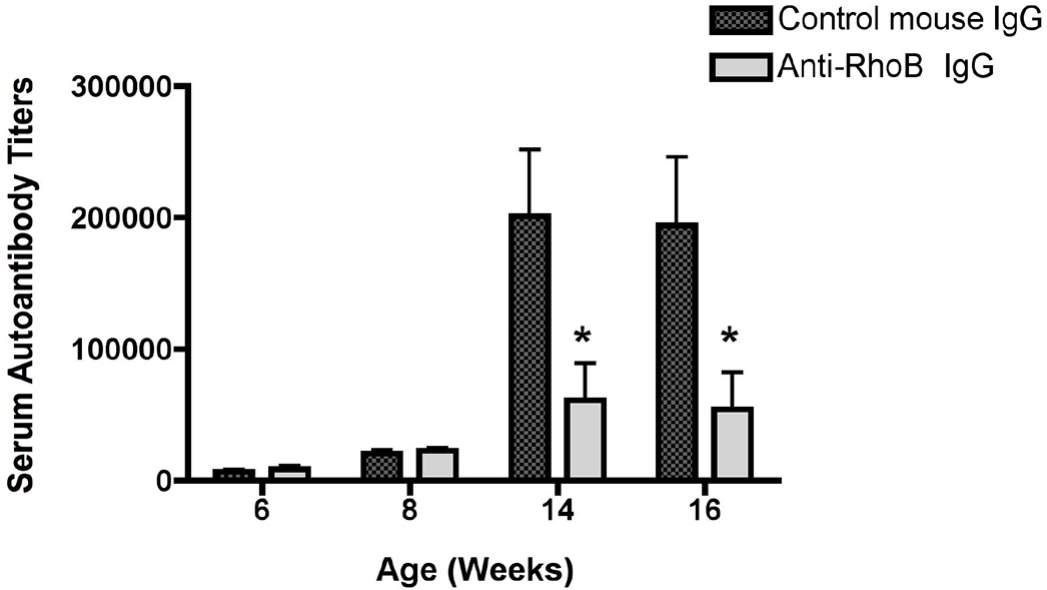
**Anti-RhoB Ig lowers autoantibody levels in the MRL/*lpr* mouse model of SLE.** Weekly doses (500 µg) of anti-RhoB IgG or control mouse IgG were administered to MRL/*lpr* mice starting at 4 weeks of age. At 16 weeks of age, serum dsDNA-autoantibody titers were determined by ELISA. Data show means±s.e.m. from two separate experiments combined for *n*=14 mice per group. Comparisons were made between control and anti-RhoB IgG groups at the specified weeks of age. *P<0.05.

To address the potential for model-specific effects to explain our observations, we determined whether administration of anti-RhoB Ig could similarly affect autoantibody levels in the MRL/*lpr* model of SLE, as a second established model of autoimmune disease. MRL/*lpr* mice develop a systemic autoimmune disease that shares many characteristics with human SLE, including high titers of autoantibodies against nuclear antigens (e.g. dsDNA) and immune-complex glomerulonephritis (Theofilopoulos and Dixon, 1985). MRL/*lpr* mice were treated with anti-RhoB Ig or control Ig starting at 4 weeks of age and followed for the development of anti-dsDNA antibodies in the serum. Similarly to untreated mice, control-Ig-treated mice developed high titers of anti-dsDNA Ig. In contrast, anti-RhoB-Ig-treated mice exhibited a significant reduction in anti-dsDNA antibody levels by 14 weeks of age (Fig. 6), confirming a role for RhoB in mediating autoantibody production in two independent models of inflammatory autoimmune disease.

## DISCUSSION

In summary, our observations using two different preclinical autoimmune models suggest a function for RhoB in specifically mediating the production of pathogenic autoantibodies, which contribute to autoimmune disease. These findings also offer preclinical proof of concept for the development and use of RhoB-directed antibodies as novel biological probes with possible therapeutic potential.

RhoB acts as a stress-response mediator (Hall, 2012; Bravo-Nuevo et al., 2011b; Huang et al., 2010), unique in the Rho family of small GTPases, and is an early-response gene encoding a short-lived protein that localizes to various vesicular membranes (Chen et al., 2006; Croft and Olson, 2011; Fritz et al., 1999, 1995; Prendergast, 2001; Wheeler and Ridley, 2007). Studies in genetically deficient cells and animals suggest broad roles in mediating Akt, Src and ERK signaling events and their subcellular localization (Adini et al., 2003; Huang et al., 2007, 2011). The complexity of RhoB-mediated signaling and compartmentalization may contribute to the difference we observed in anti-GPI titers between antibody-treated and RhoB-knockout mice. Although the endpoint of reduced disease severity was shared when RhoB was targeted by genetic deletion or with an antibody, the mechanism by which arthritis was attenuated may be different. Constitutive *RhoB* gene loss would influence the development, differentiation and maturation of B and T cells necessary for disease pathology to occur in the K/BxN model (Korganow et al., 1999; Matsumoto et al., 1999), whereas antibody targeting may affect only one or a number of these processes. Targeting RhoB function using an antibody would have a more limited contextual effect that may overlap with but not completely equate with genetic loss. Also, genetic loss of *RhoB* would eliminate RhoB enzyme activity at all subcellular locations at which RhoB is present (Adini et al., 2003; Huang et al., 2007, 2011), whereas an antibody directed to the C-terminal half of RhoB, away from the active site, may alter only the protein interactions in which RhoB participates. A scenario of misregulated enzyme function in altered subcellular locales may explain the difference in anti-GPI titer levels observed between RhoB antibody administration and genetic deficiency. More extensive work needs to be done to understand the differences of active RhoB-mediated signaling pathways and protein interactions in B and T cells when RhoB is targeted by antibody or gene loss, with an overarching goal to understand the role that RhoB plays in the adaptive antibody response as compared to autoantibody production in an autoimmune response.

Our work suggests that RhoB is a new therapeutic target for the treatment of autoantibody-mediated autoimmune diseases. Intracellular proteins such as RhoB have classically been targeted using small-molecule inhibitors; however, a growing literature suggests that a monoclonal antibody approach can also be an effective therapeutic strategy for intracellular targets. Recent reviews highlight examples of antibodies similar to the anti-RhoB antibody described here that can penetrate cells, interact with intracellular targets and therapeutically ablate disease (Hong and Zeng, 2014; Wang et al., 2015; Weidle et al., 2013). Although several mechanisms for internalization of therapeutic antibodies against intracellular targets have been suggested, it seems that no single mechanism may fully explain cellular entry (Hong and Zeng, 2014; Wang et al., 2015; Weidle et al., 2013; Choi et al., 2014; Kim et al., 2016; Merlo et al., 2017; Monteith et al., 2016). Of particular interest to our work is the study by Guo and coworkers (Guo et al., 2008) that demonstrated the anti-cancer effects of monoclonal antibodies to the phosphatases of regenerating liver (PRL) family of intracellular phosphatases. PRL proteins, similarly to RhoB, are prenylated, localized to internal cellular membranes and participate in cell signaling events to promote cell growth and migration. When antibodies to PRL1 or PRL3 were administered in murine models of metastatic tumorigenesis, tumor growth and metastatic tumor formation were blocked (Guo et al., 2008). Although the mechanism accounting for the internalization and immunotherapeutic effect is not completely understood, several factors have been identified as relevant to the process. The Fc portion of the antibody is needed for internalization (but is not isotype specific), complement is not necessarily involved, and B cells and natural killer (NK) cells are required (reviewed in Hong and Zeng, 2014). Future work is needed to compare and contrast the mechanistic factors influencing the therapeutic potential of this growing class of immunotherapeutics.

Relevant to our work, Chinestra and colleagues have reported a selective single-chain variable-fragment antibody that recognizes GTP-bound RhoB; however, the *in vivo* features of this molecule have yet to be explored (Chinestra et al., 2014). Small-molecule inhibitors of RhoB have not been described, although molecules that target RhoA, Rac and Cdc42 have been (Biro et al., 2014), including Rac inhibitors studied in models of type 1 diabetes and collagen-induced arthritis (Veluthakal et al., 2016). These studies provide evidence for important roles of Rho proteins outside of their GTPase activity and that targeting Rho effector functions *in vivo* may be feasible. The rationale behind targeting RhoB is appealing given its dispensability, unlike other Rho GTPases in the lymphoid compartment (Mulloy et al., 2010; Reedquist and Tak, 2012; Saoudi et al., 2014). These studies highlight that future work is needed to: (1) define the role of RhoB in aspects of innate and adaptive immunity, and (2) characterize aspects of RhoB-targeting approaches on potential immunosuppressive side effects that are observed with other immunotherapeutic biologics (Lateef and Petri, 2010; Singh, 2016; Tanaka, 2016; Woodworth and den Broeder, 2015). Collectively, our findings offer an early illustration of how an anti-RhoB antibody may be useful for the study and treatment of autoimmune disorders driven by the production of pathogenic autoantibodies, including RA, SLE, myasthenia gravis and type 1 diabetes.

## MATERIALS AND METHODS

### Animals

KRN T-cell-receptor transgenic mice have been described (Kouskoff et al., 1996; Monach et al., 2008). The RhoB ko mice (Liu et al., 2001) were backcrossed on to the C57BL/6 strain for 10 generations. MRL/MpJ-Fas*^lpr^* (MRL/*lpr*) and NOD mice were purchased from The Jackson Laboratory, ME, USA. To generate arthritic mice, KRN C57BL/6 mice were crossed with NOD mice, yielding KRN (C57BL/6×NOD)F_1_ mice, designated K/BxN, or with C57BL/6 mice expressing the I-A^g7^ MHC class II molecule to yield KRN C57BL/6 mice expressing I-A^b/g7^ (termed KRN.g7). To obtain arthritic RhoB ko KRN.g7 mice, RhoB ko KRN C57BL/6 mice were crossed with RhoB ko C57BL/6 mice expressing I-A^g7^ to yield RhoB ko KRN C57BL/6 I-A^b/g7^ mice (termed RhoB ko KRN.g7). All mice were bred and housed under specific pathogen-free conditions in the animal facility at the Lankenau Institute for Medical Research (LIMR; PA, USA). Overall, an approximately equal number of males and females were evaluated in various experimental protocols. In general, all pups resulting from at least three breeding pairs of KRN mice crossed to NOD or B6.g7 mice were randomly grouped for treatment or analysis with an effort to equate the number of males and females per group. Experiments shown were conducted a minimum of three times and the data from all animals analyzed was combined. Not all mice treated in a similar manner, i.e. mice treated with anti-RhoB Ig, were housed in the same cage. Males and females were caged separately and a maximum of five mice were housed in one cage. Studies were performed in accordance with the National Institutes of Health and Association for Assessment and Accreditation of Laboratory Animal Care guidelines, with approval from the LIMR Institutional Animal Care and Use Committee.

### Generation of anti-RhoB hybridomas

To address a long-standing issue in the RhoB field for generating hybridomas that could stably secrete anti-RhoB IgG, which we hypothesized was due to an autocrine blockade in antibody secretion, we employed *RhoB^–/–^* splenic cells as myeloma fusion partners in generating hybridomas. Splenocytes isolated from a *RhoB* nullizygous mouse were treated *in vitro* with lipopolysaccharide (LPS; 25 µg/ml) for 48 h. These activated B cells were then fused with the hybridoma fusion partner Sp2/0-Ag14 (American Type Culture Collection, VA, USA) using standard fusion methods. After fusion, cells were plated and isolated from methylcellulose using the vendor's protocol (Stem Cell Technologies, BC, Canada). An isolated cell line that did not secrete Ig and was heterozygotic for the *RhoB* null allele was reverted to hypoxanthine-aminopterin-thymidine (HAT) sensitivity by growth in 8-azaguanine (20 µg/ml). This new line was used in a fusion with splenocytes from a *RhoB* nullizygous 129/SJ mouse immunized in complete Freund's adjuvant with the KLH-conjugated RhoB peptide (aa140-158; accession number: NP_004031). This initial immunization was followed by a boost of the same peptide-KLH in incomplete Freund's adjuvant and then a final boost of the peptide-KLH conjugate in phosphate-buffered saline (PBS). Hybridomas were generated by standard methods, plated in methylcellulose and screened by ELISA against the unconjugated RhoB peptide (aa140-158).

### Anti-RhoB Ig treatment

Administration of Ig in the arthritis model was done by injecting mice i.p. with 0.5 mg control mouse IgG (Jackson ImmunoResearch, PA, USA; code number 015-000-003) or anti-RhoB IgG from clone 7F7 at 21 days of age, unless otherwise stated. MRL/*lpr* mice were dosed weekly from 4 to 16 weeks of age with 0.5 mg control mouse IgG or anti-RhoB IgG.

### Arthritis induction by serum transfer

Serum was collected and pooled from 8-week-old arthritic K/BxN mice or 6-week-old arthritic KRN.g7 and RhoB ko KRN.g7 mice. To induce arthritis, 150 µl of serum equalized for anti-GPI titer was injected i.p. into naive C57BL/6 or RhoB ko C57BL/6 mice on day 0. Arthritis induced by this method is transient, beginning 48 h after serum transfer and resolving 2-3 weeks later (Korganow et al., 1999).

### Arthritis incidence

The two rear ankles of K/BxN, KRN.g7 or RhoB ko KRN.g7 mice were measured starting at weaning (3 weeks of age). Measurement of ankle thickness was made above the footpad axially across the ankle joint using a Fowler Metric Pocket Thickness Gauge. Ankle thickness was rounded off to the nearest 0.05 mm. At the termination of the experiment, ankles were fixed in 10% buffered formalin for 48 h, decalcified in 14% EDTA for 2 weeks, embedded in paraffin, sectioned and stained with H&E. Histological sections were imaged using a Zeiss Axioplan microscope with a Zeiss Plan-Apochromat 10×/0.32 objective and Zeiss AxioCam HRC camera using AxioVision 4.7.1 software. The images were then processed using Adobe Photoshop CS2 software.

### Immunization with model antigen NP

C57BL/6 or RhoB ko C57BL/6 mice were immunized i.p. with 100 µg NP-KLH (Biosearch Technologies, CA, USA) precipitated in alum as described previously (Jacob et al., 1991). The mice were sacrificed 2 weeks later and serum harvested for anti-NP Ig titers.

### ELISA

#### Anti-GPI ELISA

Serum samples were plated at an initial dilution of 1:100 and diluted serially 1:5 in Immulon II plates coated with recombinant GPI-his (10 µg/ml). The serum titer was defined as the reciprocal of the last dilution that gave an OD>3× background.

#### Anti-Ig ELISA

Serum samples were plated at an initial dilution of 1:100 and diluted serially 1:5 on Immulon II plates coated with unlabeled donkey anti-mouse total Ig (Jackson ImmunoResearch). Purified mouse IgM, IgG_1_, IgG_2a_, IgG_2b_ and IgG_3_ (Southern Biotechnology Associates, AL, USA) were used to generate standard curves. The total amount of Ig in the serum was calculated from the standard curve using Prism 4 software (GraphPad Software, Inc., CA, USA).

#### Anti-NP ELISA

Serum samples were plated at an initial dilution of 1:100 and diluted serially 1:4 on Immulon II plates coated with 2 µg/ml NP_3_-BSA or NP_16_-BSA (Biosearch Technologies). The serum titer was defined as the reciprocal of the last dilution that gave an OD>3× background.

#### Anti-dsDNA ELISA

Serum samples were plated at an initial dilution of 1:100 and diluted serially 1:4 on Immulon II plates coated with poly-L-lysine and dsDNA. The serum titer was defined as the reciprocal of the last dilution that gave an OD>3× background.

#### For all ELISAs

Goat anti-mouse total IgG_Fc_-HRP (Jackson ImmunoResearch), goat anti-mouse IgM-HRP, IgG_1_-HRP, IgG_2a_-HRP, IgG_2b_-HRP, IgG_2c_-HRP or IgG_3_-HRP (Southern Biotechnology Associates) were used as secondary antibodies (dilutions 1:1000). Antibody was detected using ABTS substrate (Fisher Scientific, NH, USA).

### ELISpot assay

Cells from the joint-draining LNs (axillary, brachial and popliteal) were plated at 4×10^5^ cells per well and diluted serially 1:4 in MultiScreen-HA mixed-cellulose-ester membrane plates (Millipore, CA, USA) coated with GPI-his (10 µg/ml). The cells were incubated on the antigen-coated plates for 4 h at 37°C. The Ig secreted by the plated cells was detected by alkaline-phosphatase-conjugated goat anti-mouse total Ig secondary antibody (Southern Biotechnology Associates) and visualized using NBT/BCIP substrate (nitroblue tetrazolium/5-bromo-4-chloro-3-indolyl phosphate; Sigma-Aldrich, MO, USA).

### Cytokine secretion

Cells from the joint-draining LNs were harvested and cultured (2×10^6^/ml) in PMA (50 ng/ml)+ionomycin (500 ng/ml) for 24 h. The supernatants were then harvested and analyzed for the levels of cytokines by cytometric bead array (BD Biosciences, CA, USA). The samples were stained according to manufacturer instructions and analyzed on a FACSCanto II flow cytometer (BD Biosciences) using FACSDiva software (BD Biosciences). Cytokine concentrations were calculated by comparing to standard curves using FCAP array analysis software (BD Biosciences).

### Flow cytometry

A total of 1×10^6^ bone marrow, thymus, spleen, LN or peritoneal cavity cells were stained with antibodies to CD3 (145-2C11), CD5 (53-7.3), CD8α (53-6.7), B220 (RA3-6B2), IgM (RMM-1), IgD (11-26.2a) (BioLegend, CA, USA); CD4 (GK1.5), CD23 (B3B4), CD25 (PC61.5) (eBioscience, CA, USA); CD21/35 (7G6) and CD43 (S7) (BD Biosciences) using dilutions of 1:100 or 1:200. Samples were analyzed on a FACSCanto II flow cytometer using FACSDIVA software. Data were analyzed using FlowJo software (TreeStar, OR, USA). Gating on live lymphocytes was based on forward and side scatter, with 50,000 events collected for each sample. Immune-cell subsets were defined based on cell surface markers.

### Western blot analysis

Tissue was harvested and homogenized in a Polytron blender in the presence of RIPA buffer containing protease and phosphatase inhibitors. Tissue lysates were centrifuged and protein concentrations determined. Recombinant proteins RhoB (OPPA00123), RhoA (OPPA00043), Rac1 (OPPA01858) and Cdc42 (OPPA00141) were purchased from Aviva Systems Biology (San Diego, CA, USA). Tissue extract (50 µg tissue extract/lane) or recombinant protein (5 or 20 ng/lane) was fractioned using standard SDS-PAGE and blotted to Immobilon-NC membranes (Millipore). After blocking, the blots were incubated at 4°C overnight with primary antibody, followed by incubation with an HRP-conjugated secondary antibody (antibody dilutions 1:500-1:1000). Blots were washed, developed with HYGLO Quickspray chemiluminescent HRP reagent (Denville Scientific, NJ, USA) and analyzed using a ChemiDoc System with Image Lab software (Bio-Rad, CA, USA). Primary antibodies to the following antigens were used: RhoB (7F7), Cdc42 (SC-8401; Santa Cruz Biotechnology, CA, USA); RhoA (SC-418; Santa Cruz Biotechnology); and Rac1 (05-389, EMD Millipore). Appropriate HRP-conjugated secondary antibodies were used for each primary antibody.

### Statistical analysis

Statistical significance was determined using an unpaired Student's *t*-test when comparing arthritis at specified time points, and the Mann–Whitney nonparametric test when comparing ELISA or ELISpot data and Prism Software (GraphPad Software, Inc., CA, USA).

## Supplementary Material

Supplementary information
