# Supplementary material for: RhoB blockade selectively inhibits autoantibody production in autoimmune models of rheumatoid arthritis and lupus
Source: Dis Model Mech. 2017 Nov 1;10(11):1313–22. doi: 10.1242/dmm.029835 (PMC5719251; doi:10.1242/dmm.029835)
Supplement: Supplementary information [file dmm-10-029835-s1.pdf]

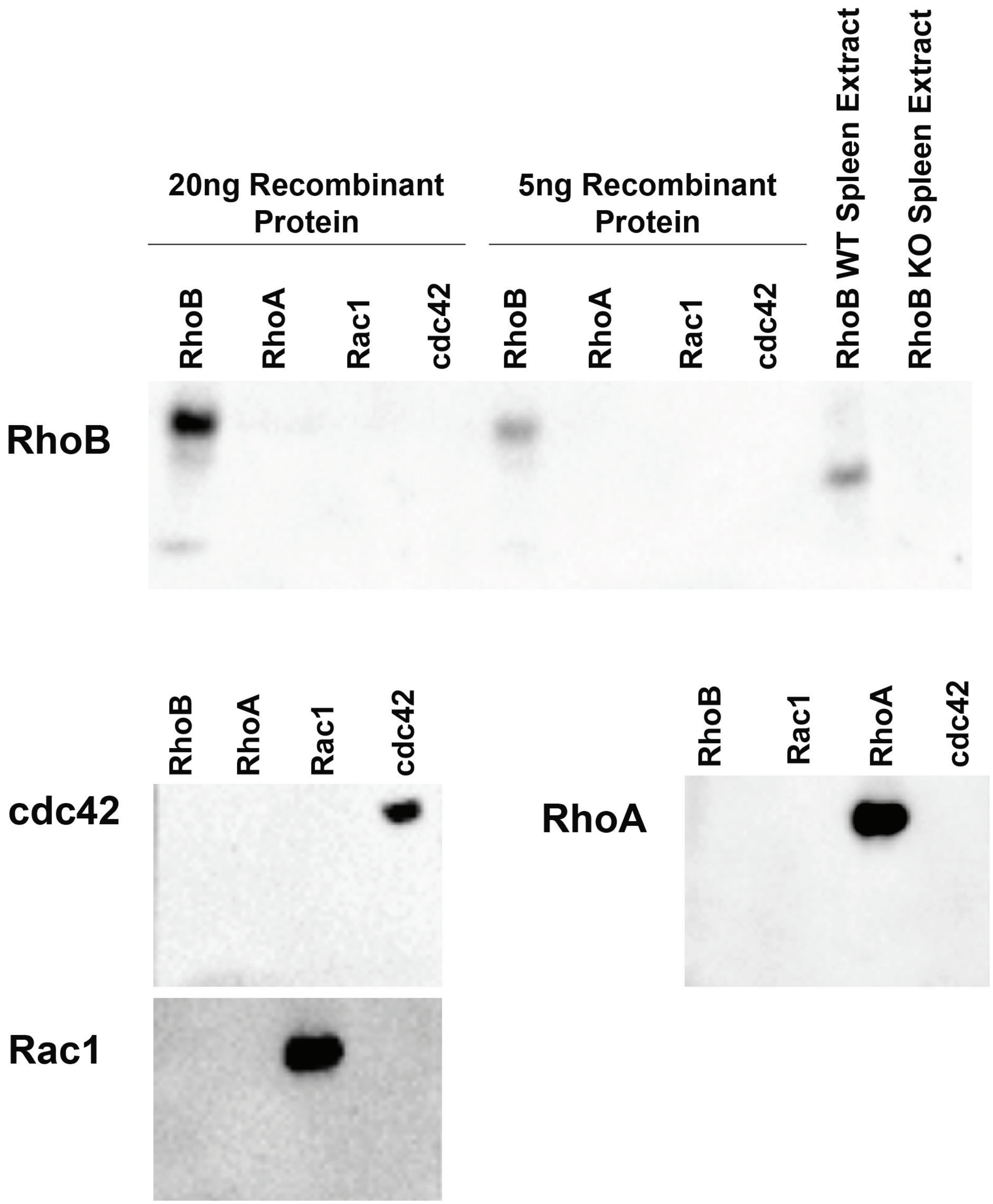

Supplemental Figure 1. The anti-RhoB IgG is specific for RhoB, not other RhoGTPase family members. The anti-RhoB antibody generated in our laboratory was used with standard western blotting protocols against recombinant RhoB, RhoA, Rac1 and cdc42 proteins, indicated across the top of the blots. Extracts from wildtype (WT) or RhoB (KO) C57BL/6 splenic tissue are shown as positive and negative controls. Companion blots were probed with commercial anti-RhoA, Rac1 or cdc42 antibodies for comparison. The antibody used on the western blot is indicated on the left. Data shown is representative from two separate analyses.

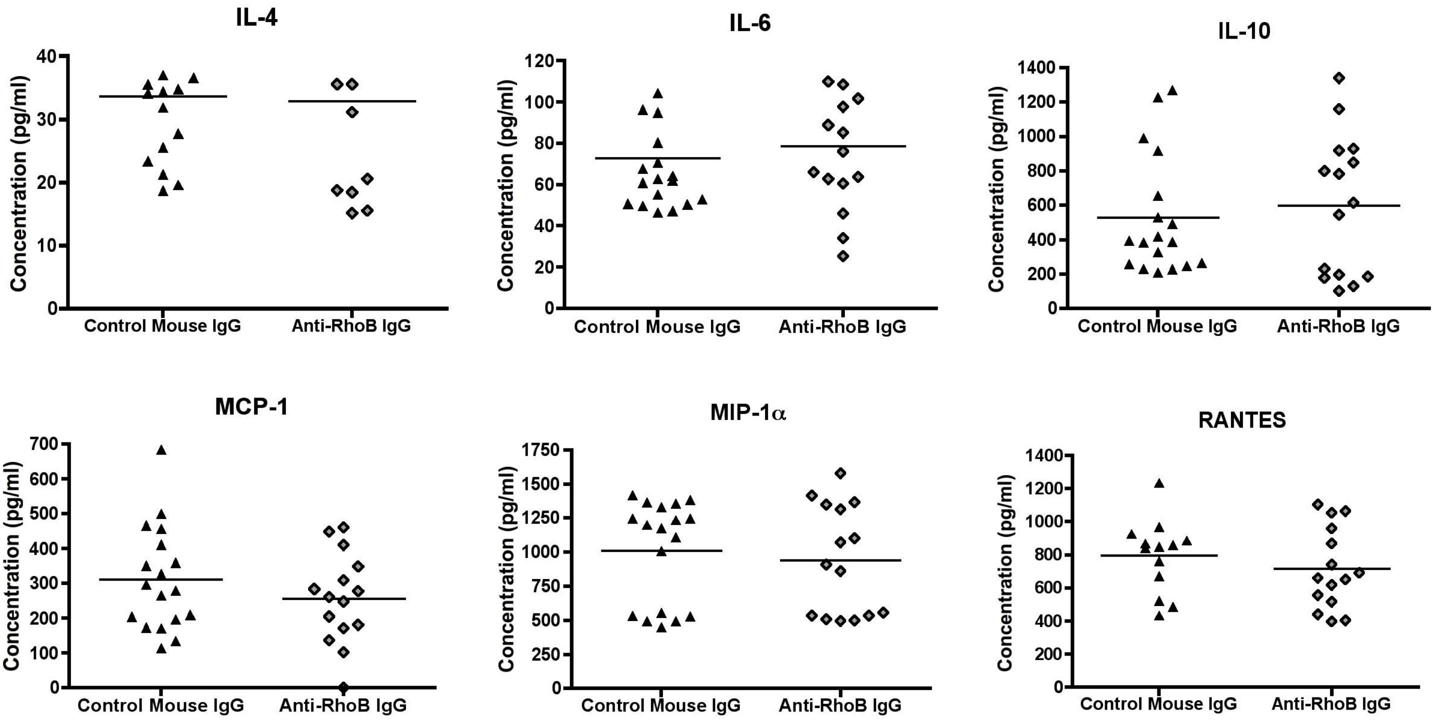

Supplemental Figure 2. Anti-RhoB Ig does not affect inflammatory cytokines. K/BxN mice were treated with 500 µg anti-RhoB Ig at 21 days of age. Three weeks later, the lymph nodes draining the arthritic joints were harvested and stimulated with PMA + Ionomycin overnight. Inflammatory cytokines were measured in the supernatants by cytometric bead array using flow cytometry. Each symbol depicts an individual mouse with the mean indicated by a solid bar (▲Control Ig, n=18; ◇anti-RhoB Ig, n=15).

Disease Models & Mechanisms • Supplementary information

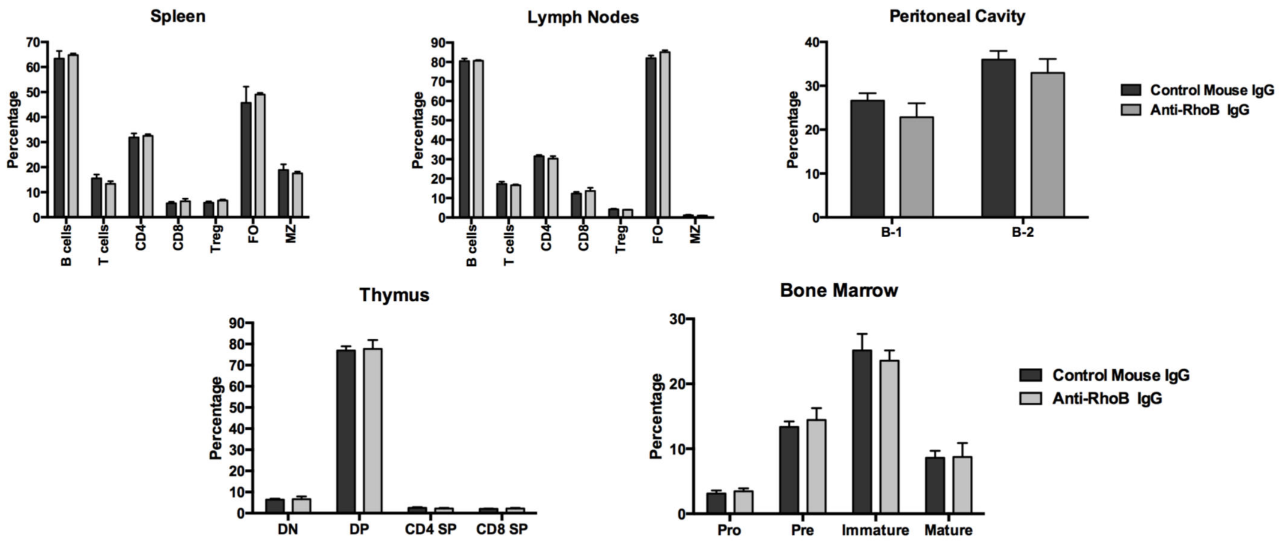

Supplemental Figure 3. Anti-RhoB Ig does not affect the immune cell repertoire in K/BxN mice. The percentage of individual lymphoid populations and serum anti-GPI titers were determined in K/BxN mice. Control mouse IgG or anti-RhoB IgG was administered once at 3 weeks of age and experiment terminated at 6 weeks of age. The frequency of individual lymphoid populations in control Ig or anti-RhoB Ig in K/BxN mice was measured by flow cytometry. Data show mean ± SEM from a representative experiment of two with n= 10-12 mice for each group.

Disease Models & Mechanisms • Supplementary information

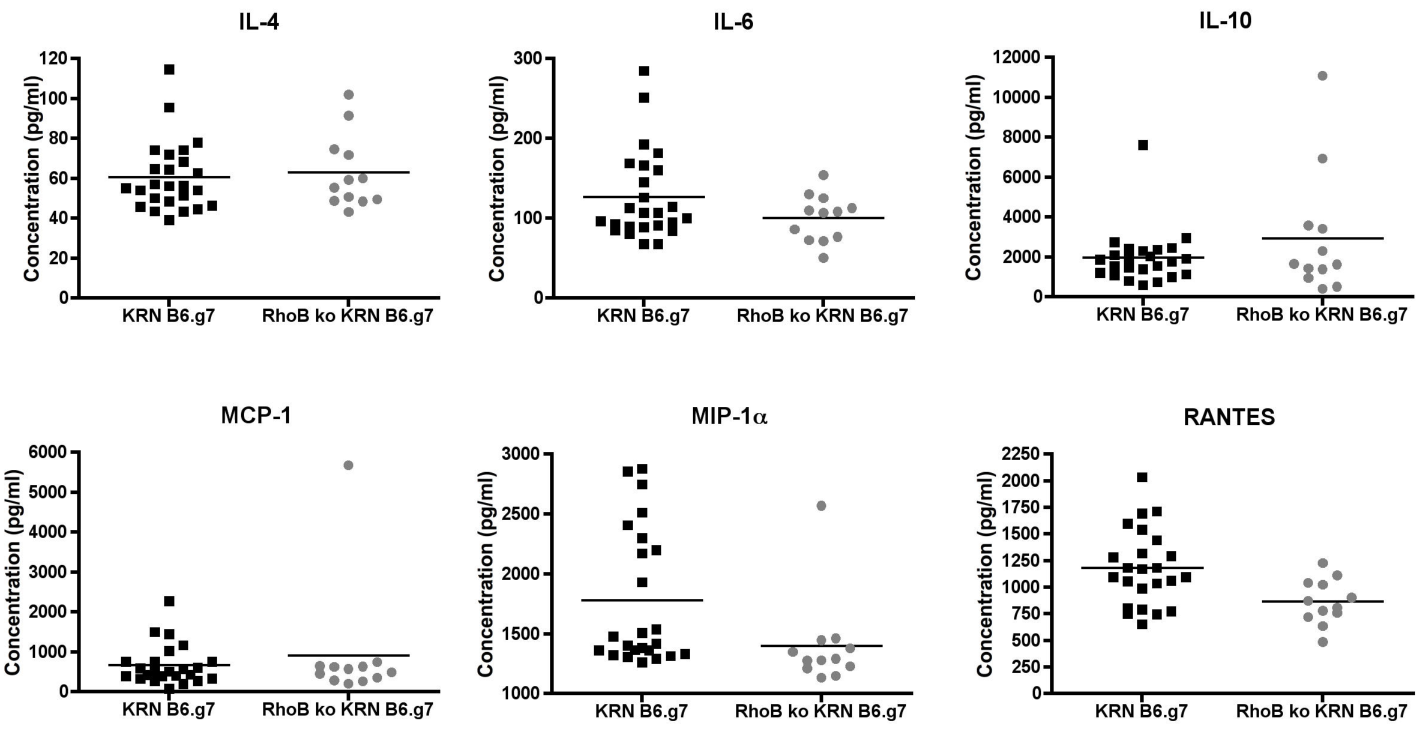

Supplementary Figure 4. Inflammatory cytokines are not altered in RhoB ko arthritic mice. At 6 wks of age, the lymph nodes draining the arthritic joints were harvested and stimulated with PMA + Ionomycin overnight. Inflammatory cytokines were measured in the supernatants by cytometric bead array using flow cytometry. Each symbol depicts an individual mouse with the mean indicated by a solid bar (■ KRN B6.g7, n=25; ● RhoB ko KRN B6.g7, n=12).

Disease Models & Mechanisms • Supplementary information

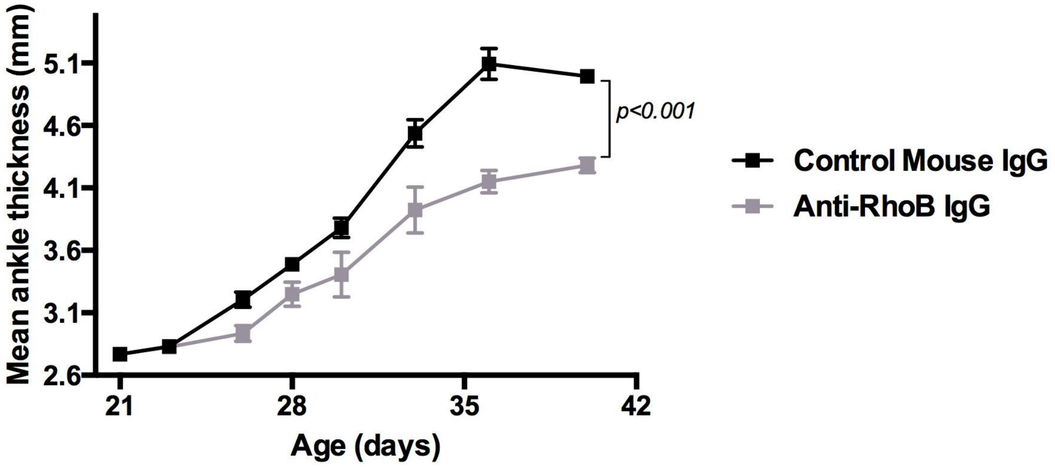

Supplemental Figure 5. Anti-RhoB Ig attenuates arthritis in wildtype KRN.g7 mice. Joint inflammation was determined by measuring ankle thickness. Data show mean ± SEM from a representative experiment of two with n= 4-5 mice for each group. ■wt KRN B6.g7 dosed with Ig control, ■wt KRN B6.g7 dosed with anti-RhoB Ig. Data shown is representative from two separate analyses.

Disease Models & Mechanisms • Supplementary information

S6A

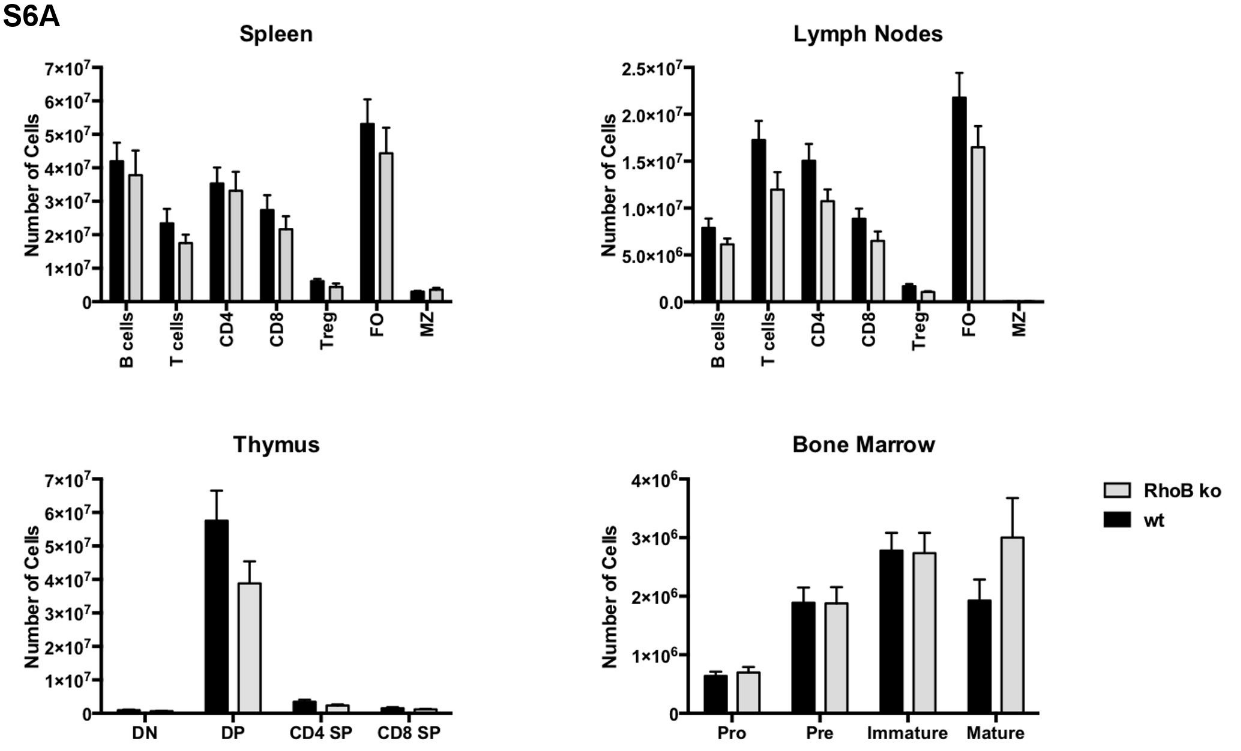
Spleen
Lymph Nodes
Thymus
Bone Marrow
Number of Cells
B cells
T cells
CD4
CD8
Treg
FO
MZ
DN
DP
CD4 SP
CD8 SP
Pro
Pre
Immature
Mature
RhoB ko
wt

S6B

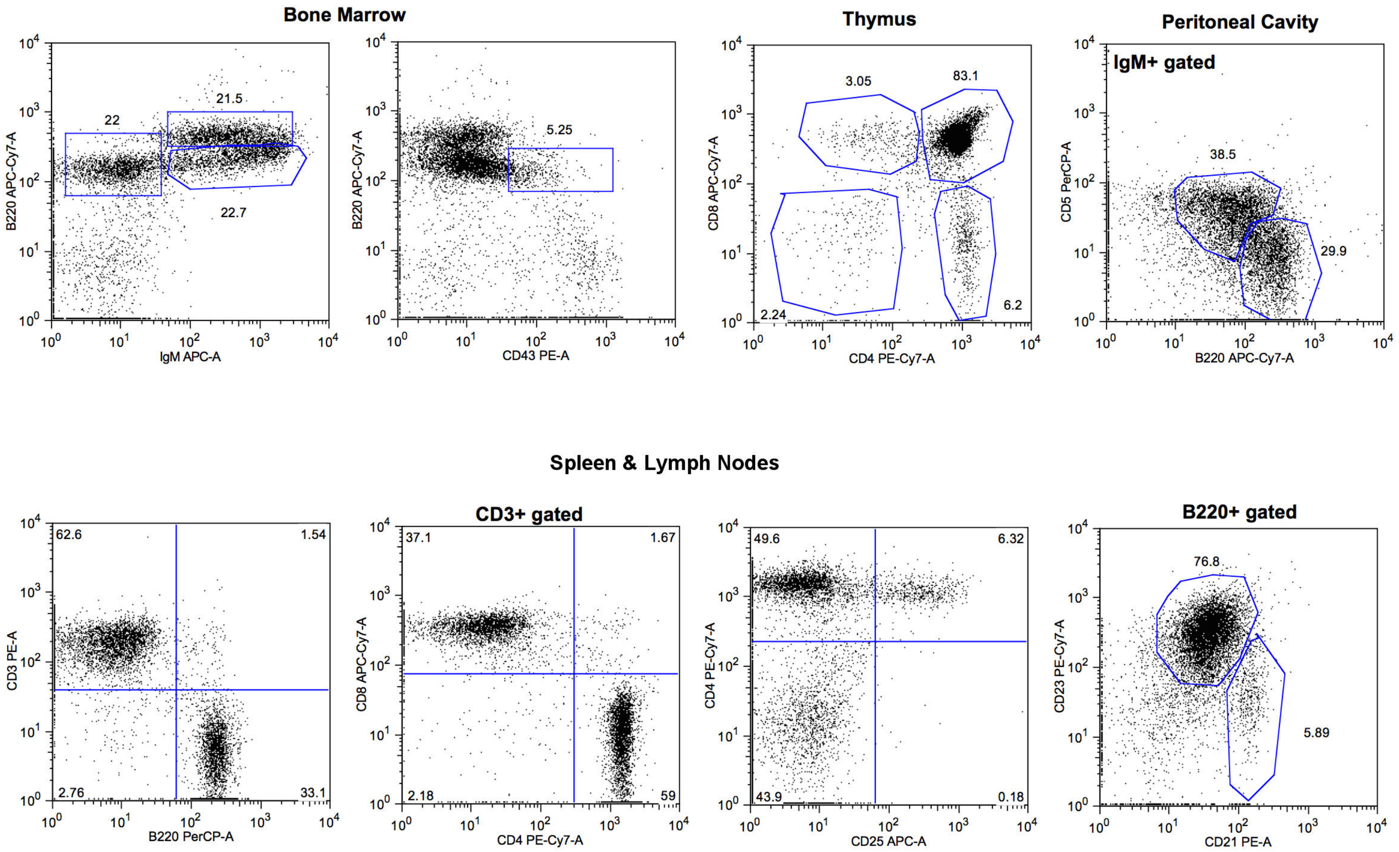
Bone Marrow
Thymus
Peritoneal Cavity
IgM+ gated
B220 APC-Cy7-A
IgM APC-A
CD43 PE-A
CD8 APC-Cy7-A
CD4 PE-Cy7-A
CD5 PerCP-A
Spleen & Lymph Nodes
CD3+ gated
B220+ gated
CD3 PE-A
B220 PerCP-A
CD4 PE-Cy7-A
CD25 APC-A
CD23 PE-Cy7-A
CD21 PE-A

Supplemental Figure 6. Targeting RhoB does not affect the absolute numbers of the lymphoid populations in either wt or RhoB ko B6 mice. (a) The absolute number of individual lymphoid populations in wt or RhoB ko C57BL/6 mice was measured by flow cytometry. n=6 mice of each genotype. This data corresponds to the data presented in Figure 5a. Data show mean ± SEM. All analyses were performed 3 times. (b) The flow cytometry gating strategies used to determine the lymphoid cell frequencies and absolute numbers throughout this study. Lymphoid populations were defined as follows. Bone Marrow: ProB ($B220^{low}IgM^{-}CD43^{+}$), PreB ($B220^{low}IgM^{-}CD43^{-}$), Immature B ($B220^{low}IgM^{+}$), Mature B ($B220^{high}IgM^{+}$); Thymus: DN ($CD4^{-}CD8^{-}$), DP ($CD4^{+}CD8^{+}$), CD4 SP ($CD4^{+}CD8^{-}$), CD8 SP ($CD4^{-}CD8^{+}$); Peritoneal Cavity: B-1 ($B220^{+}CD5^{+}$), B-2 ($B220^{+}CD5^{-}$); Spleen and LN: B ($B220^{+}$), T ($CD3^{+}$), CD4 ($CD3^{+}CD4^{+}$), CD8 ($CD3^{+}CD8^{+}$), Treg ($CD3^{+}CD4^{+}CD25^{+}$), FO ($B220^{+}CD21^{low}CD23^{high}$), MZ ($B220^{+}CD21^{high}CD23^{low}$).

Disease Models & Mechanisms • Supplementary information
